# Role of Amide Proton Transfer Weighted MRI in Predicting MGMTp Methylation Status, p53-Status, Ki-67 Index, IDH-Status, and ATRX Expression in WHO Grade 4 High Grade Glioma

**DOI:** 10.3390/tomography11060064

**Published:** 2025-05-31

**Authors:** Faris Durmo, Jimmy Lätt, Anna Rydelius, Elisabet Englund, Tim Salomonsson, Patrick Liebig, Johan Bengzon, Peter C. M. van Zijl, Linda Knutsson, Pia C. Sundgren

**Affiliations:** 1 Department of Clinical Sciences/Division of Radiology, Lund University, SE-221 00 Lund, Sweden; faris.durmo@med.lu.se (F.D.); tim.salomonsson@med.lu.se (T.S.); 2 Department of Medical Imaging and Physiology, Skåne University Hospital, SE-221 85 Lund, Sweden; jimmy.latt@med.lu.se; 3 Department of Clinical Sciences/Division of Neurology, Lund University, SE-221 00 Lund, Sweden; anna.rydelius@med.lu.se; 4 Department of Clinical Sciences/Division of Pathology, Lund University, SE-221 00 Lund, Sweden; elisabet.englund@med.lu.se; 5 Advanced Systems, Magnetic Resonance, G, 91052 Erlangen, Germany; patrick.liebig@siemens-healthineers.com; 6 Department of Clinical Sciences/Division of Neurosurgery, Kamprad Laboratory, Lund University, SE-221 00 Lund, Sweden; johan.bengzon@med.lu.se; 7 Russell H. Morgan Department of Radiology and Radiological Science, Johns Hopkins University School of Medicine, Baltimore, MD 21205, USA; pvanzijl@jhu.edu; 8 F.M. Kirby Research Center for Functional Brain Imaging, Kennedy Krieger Institute, Baltimore, MD 21205, USA; linda.knutsson@med.lu.se; 9 Department of Medical Radiation Physics, Lund University, SE-221 00 Lund, Sweden; 10 Department of Neurology, Johns Hopkins University School of Medicine, Baltimore, MD 21205, USA; 11 LBIC, Lund University Bioimaging Center, Lund University, SE-221 00 Lund, Sweden

**Keywords:** amide proton transfer weighted imaging, glioma, glioblastoma, ATRX, IDH, p53, Ki-67, MGMT

## Abstract

**Objectives:** To assess amide proton transfer weighted (APTw) MR imaging capabilities in differentiating high-grade glial tumors across alpha-thalassemia/mental retardation X-linked (ATRX) expression, tumor-suppressor protein p53 expression (p53), O6-methylguanine-DNA methyltransferase promoter (MGMTp) methylation, isocitrate dehydrogenase (IDH) status, and proliferation marker Ki-67 (Ki-67 index) as a preoperative diagnostic aid. **Material & Methods:** A total of 42 high-grade glioma WHO grade 4 (HGG) patients were evaluated prospectively (30 males and 12 females). All patients were examined using conventional MRI, including the following: T1w-MPRAGE pre- and post-contrast administration, conventional T2w and 3D FLAIR, and APTw imaging with a 3T MR scanner. Receiver operating characteristic (ROC) curves were calculated for the APTw% mean, median, and max signal for the different molecular biomarkers. A logistic regression model was constructed for combined mean and median APTw% signals for p53 expression. **Results:** The whole-tumor max APTw% signal could significantly differentiate MGMTp from non-MGMTp HGG, *p* = 0.035. A cutoff of 4.28% max APTw% signal yielded AUC (area under the curve) = 0.702, with 70.6% sensitivity and 66.7% specificity. The mean/median APTw% signals differed significantly in p53 normal versus p53-overexpressed HGG s: 1.81%/1.83% vs. 1.15%/1.18%, *p* = 0.002/0.006, respectively. Cutoffs of 1.25%/1.33% for the mean/median APTw% signals yielded AUCs of 0.786/0.757, sensitivities of 76.9%/76.9%, and specificities of 50%/66.2%, *p* = 0.002/0.006, respectively. A logistic regression model with a combined mean and median APTw% signal for p53 status yielded an AUC = 0.788 and 76.9% sensitivity and 66.2% specificity. ATRX-, IDH- wild type (wt) vs. mutation (mut), and the level of Ki-67 did not differ significantly, but trends were found: IDH-wt and low Ki-67 showed higher mean/median/max APTw% signals vs. IDH-mut and high Ki-67, respectively. ATRX-wt vs. mutation showed higher mean and median APTw% signals but lower max APTw% signal. **Conclusions**: APTw imaging can potentially be a useful marker for the stratification of p53 expression and MGMT status in high-grade glioma in the preoperative setting and potentially aid surgical decision-making.

## 1. Introduction

Despite maximal surgical resection and concomitant/subsequent radio-chemotherapy, malignant gliomas continue to have a very poor prognosis. The median survival in its most common and most malignant form, glioblastoma grade 4, is 15 months overall survival (OS) and the median progression-free survival (PFS) with standard treatment is 7 months [1,2,3,4,5]. The loss of molecular integrity plays a key role in the genesis of glioma. Tumor molecular profiling is central for prognosis and treatment planning [6], mirrored in the WHO 2016 classifications, where isocitrate dehydrogenase 1 and 2 (IDH1/2) mutations and alpha thalassemia/mental retardation syndrome X-linked (ATRX), among others, were incorporated as diagnostic criteria for tumors of glial origin [6,7,8]. This was further stratified in the latest WHO Classification of CNS Tumors 2021 (WHO CNS5/2021), in which only IDH wild type tumors (IDHwt) are classified as glioblastoma grade 4 (GBM) [5].

Alterations in enzymes encoded by mutated IDH genes impact cellular metabolism through disturbances in the normal operation of the citric acid cycle within the cellular cytosol [9]. These disturbances range from shifts in enzymatic activity to the down- or upregulation of amino acid concentrations [9,10,11,12,13]. Heterozygous missense mutations of arginine to histidine, R132H, make up more than 85% of the genetic alterations in IDH1-mutated gliomas. The presence of IDH mutations (IDH-mut) has been shown to be positively associated with mutations of ATRX [14,15,16].

The product of the TP53 tumor suppressor gene, p53, is accumulated in transformed cells in most cancer types. It acts through transcriptional activity in the cell cycle leading to cell cycle arrest [17]. In the majority of high-grade gliomas (HGGs), the TP53 gene is not mutated [8]. However, in GBM cell lines, p53 expression has been shown to impact the level of cell invasion, proliferation and migration, as well as the ability of the cell to escape apoptosis [18]. This implies that p53 might act as a biomarker when following treatment effects in certain types of cancer with either chemotherapy [19,20] or radiation [20,21], as well as being a potential target for future therapy [22].

The protein expression of the Ki-67 antigen is known to be associated with cell proliferation and is present during all cell cycle phases [23]. It has been shown that the Ki-67 index is elevated in higher malignancy gliomas [24] and that a higher APTw% signal correlates positively with the Ki-67 index [25].

The O^6^-methylguanine-DNA methyltransferase (MGMT) promoter methylation is associated with a higher degree of survival in high-grade glioma, partly due to better treatment response to alkylating agents [9,26,27]. MGMT promoter (MGMTp) methylation downregulates the protein synthesis of the MGMT protein and, by proxy, DNA repair, which enables alkylating agents to exert more damage to the malignant glioma cells [9,26,27]. The consensus is that MGMT gene silencing, i.e., the methylation of the gene epigenetically, provides longer survival due to a decreased ability to repair the damaged genome of malignant cells [9,26,27,28]. MGMTp methylated glioblastomas respond better to temozolomide, with the opposite being true for unmethylated high-grade gliomas [29].

As more evidence is accumulated on ATRX alterations in IDH-mutated gliomas [30], MGMTp methylation in general [28], and their impact on survival and progression in high-grade glioma patients, an increasing demand arises for studies elucidating the relationship between genetic mutations and improved early diagnosis. The most malignant form of malignant gliomas, GBM, has been classified and re-classified over the years depending on their molecular characteristics [5,7,31]. GBM consists of heterogeneous cell states with high plasticity and dynamic tumor evolution and co-evolution of the microenvironment [32,33]. Different stressors such as hypoxia, ischemia, and treatments impact GBMs to activate different transcriptional factors [32] where the tumor cells not only modify their own phenotype but also that of the microenvironment adjacent to gadolinium (Gd) T1-weighted enhancing areas in the brain [32,34]. Hypoxia and lactate production contribute to GBM aggressiveness, as a reduction in extracellular extravascular pH mediates invasive growth [35,36,37,38]. Reflecting this, the outer tumor rim adjacent to the normal parenchyma has an increased Ki-67 index [36,38].

Amide proton transfer weighted (APTw) MRI is a chemical exchange saturation transfer (CEST) technique in which the amide protons in mobile cellular proteins and peptides can be indirectly assessed through the water signal, making the technique promising for the noninvasive assessment of brain tumors [39,40]. APTw% signal has been shown to correlate strongly with cell density in the peritumoral environment [41], besting arterial spin labeling in predicting tumor invasion [41,42]. It has been shown that APTw MRI allows for the differentiation of high- and low-grade gliomas with comparable accuracy to dynamic susceptibility contrast perfusion weighted (DSC PW) MRI [42,43]. In addition, the technique has the capability to differentiate between pseudo progression and true progression [44,45,46]. Following the inclusion of molecular markers in the WHO 2016 classification, APTw MRI has been investigated as an imaging biomarker technique with studies providing evidence on the feasibility of predicting IDH1/2 status [47,48,49,50,51]. However, there are still inconclusive or contradictory results on whether APTw MRI can predict MGMTp methylation status [47,48,52,53,54] and as mentioned, the tumor classifications have been further revised with WHO CNS5/2021 [5]. Therefore, there is a need for additional studies to evaluate the relationship between molecular markers and APTw MRI [55]. Thus, this study aimed to assess the ability of APTw MRI to noninvasively differentiate malignant, high-grade glioma grade 4 as defined by the WHO 2016 and the WHO CNS5/2021 classifications in terms of ATRX expression, MGMTp methylation status, IDH status, Ki-67 index and p53 status.

## 2. Materials and Methods

### 2.1. Patients

The cohort consisted of 42 patients (30/12 male/female) who were prospectively included during the period of 2017–2021 for the evaluation of a suspected brain tumor. A study on a subset of 10 of these patients was included in a different study, where the APTw% signal was compared with the signal in patients with solitary CNS metastases [56]. The patients were characterized and treated according to the WHO 2016 classification. In addition, as inclusion criteria, all patients had to have both MRI imaging, including APTw imaging, and adequate molecular characterization by the neuropathologist to be included. Other inclusion criteria were a minimum age of 18 years, previous pre-operative routine MRI revealing suspected brain neoplasm, and histopathology-confirmed high-grade glioma (HGG) defined as glioblastoma grade 4 according to the criteria of WHO 2016 at time of inclusion, and according to WHO CNS5/2021 after re-classification. The overall average age was 56 years, with 56 vs. 58 years for males/females (Table 1). Written informed consent was obtained from each participant, and the project was approved by the national ethical review board (#2016/531, #2017/866, and #2018/993).

### 2.2. MRI Acquisition Protocol for APTw and Conventional MRI Sequences

Patients were examined on a 3T scanner (MAGNETOM Prisma, Siemens Healthineers, Forchheim, Germany). Conventional MR sequences included a T1-MPRAGE: TR/TE 1900/2.54 ms, FOV 256 × 256 mm^2^, acq. matrix 256 × 256, inversion time 900 ms, TA 5:13 min pre- and post-contrast administration, a FLAIR (3D): TR/TE 5000/393 ms, FOV 256 × 256 mm^2^, acq. matrix 256 × 256, inversion time 1800 ms, TA 4:25 min, T2w TSE: TRA, and TE/TR: 100/6000 ms/ms.

A vendor-supplied research CEST 3D GRE MRI sequence was used to acquire APTw images with 24 slices (4 mm thickness) and 2 × 2 mm^2^ in-plane resolution. B1 was set to 2 µT and 5 hyperbolic secant pulses (100 ms each) with 4 interpulse delays (61 ms) were applied. Water saturation spectral acquisition (Z-spectrum) was performed with saturation applied at 21 frequency offsets ±610 Hz (~ ±5ppm) along with an unsaturated reference image, S_0_, acquired at an offset of −150 ppm relative to water to avoid interference from the semisolid macromolecules (i.e., magnetization transfer effects). APTw imaging was performed before the conventional Gd-based MRI protocol as Gd reduces T1, thus quenching the APTw contrast. All images were acquired during the same session. The total scan time for the protocol was approximately 45 min, with the APTw MRI having an acquisition time of 6 min and 50 s.

### 2.3. Postprocessing of the APTw MRI Protocol

The Siemens prototype software at the scanner automatically calculated the APTw% signal in percent for each voxel. This was done by first normalizing the water signal intensities (S_sat_) at the different frequency offsets to the signal without saturation (S_0_) to obtain the so-called Z-spectrum [57]. B0 correction of the Z-spectrum was then performed by centering the direct saturation water signal at 0 ppm. Thereafter, an integral, centered at 3.5 ppm with a range of ±0.4 ppm, was calculated. A similar integral was calculated at the frequency range opposite to the water resonance (located at 0 ppm), and thus centered at −3.5 ppm. By taking the difference of these integrals (employing asymmetry analysis), the APTw% signal is obtained [34]:

$$\begin{array}{cc} APTw={MTR}_{asym}^{Integr} & \left(3.5\pm 0.4ppm\right)\\ = & \frac{\left\{P\left(-3.5\pm 0.4ppm\right)-P\left(+3.5\pm 0.4ppm\right)\right\}}{Deltafreq\cdot S_{0}}= \end{array}$$

$$=\left[C\left(x,y\right)-2048\right]/(scalefactor\cdot 2000)$$

where *C(x, y*) is the signal in a voxel and P is the summation of *S_sat_/S_0_* values over the range Deltafreq (integral range) of 0.8 ppm in the Z-spectrum. The scalefactor was set to 10. This ratio was transformed into a percentage by multiplying by 100% to obtain the APTw% signal. An MR physicist, blinded to histopathological diagnosis, prepared and co-registered the images. The APTw and FLAIR images were co-registered to the morphological MPRAGE volume using FMRIB’s linear registration tool, FLIRT, v5.0.6, Oxford, UK, [58]. Prior to co-registration, all images were masked with the FSL Brain Extraction Tool (BET v2.1) [59]. Manual segmentations were performed using 3D Slicer version 4.11.2 accessed on 1 February 2021 (http://www.slicer.org/) [60].

All slices available were assessed for the presence of tumor and pathological appearing tissue was localized and segmented by a radiologist in training (FD) under the supervision of an experienced, 20+ years neuroradiologist (PCS). The entire enhancing volume was localized based on Gd-enhanced T1-MPRAGE and FLAIR MRI, i.e., enhancing solid tumor lesion or peripheral enhancing tumor rim and peritumoral hyperintensity, respectively. Highly suspicious areas were identified and removed with assistance from apparent diffusion coefficient (ADC), perfusion weighted, Gd-enhanced T1-MPRAGE, and T2/FLAIR images accordingly to the following:

1. Necrotic regions, defined as non-enhancing cores with elevated ADC and low perfusion.
2. Cystic areas, characterized by CSF-like signal on FLAIR/T1-MPRAGE with no enhancement and elevated ADC values.
3. Major vessels, recognized by flow voids in T2-weighted images and focal perfusion intensities corresponding to arterial anatomy.

**Figure 1:**
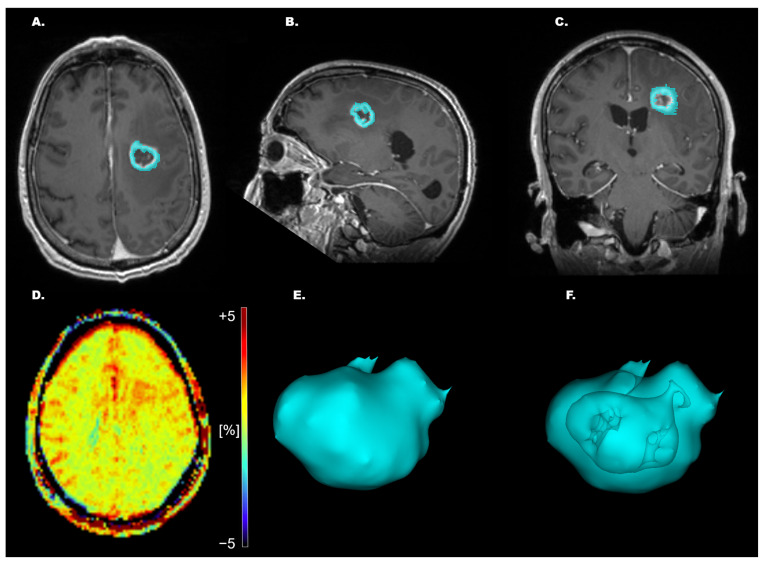
Example of whole-tumor segmentation for a high-grade glioma (HGG), with corresponding 3D rendering. (**A**–**C**) T1-MPRAGE Gd-enhanced axial/sagittal/coronal plane. (**D**) APTw image showing hotspots of increased APTw% signal from −5 to +5%. (**E**) 3D rendering of entire segmentation. (**F**) 3D rendering of entire segmentation with depiction of excluded central necrotic, cystic, or hemorrhagic areas.

These non-tumoral portions were removed from the ROI in 3D Slicer so that only viable, enhancing tumor tissue remained for APTw quantification (Figure 1). Mean, median, and maximum (max) APTw% signals were then extracted from the segmented pathological tissue.

### 2.4. Histopathology and Molecular Characterization

The evaluation and preparation of the specimens were performed by fixation in neutrally buffered formaldehyde solution (4%) and subsequently embedded in paraffin, sectioned at 4 μm and stained with routine and immunohistochemical methods. The retrospective collection of histopathological and molecular data was obtained for every patient. Medical records were electronically accessed. ATRX expression (ATRX-mutation (mut), ATRX wild type (ATRX-wt), and Ki-67 index (Ki-67 low, the specimens with Ki-67 less or equal to 10%, and Ki-67 high, specimens with Ki-67 higher than 10%), and p53 expression (defined as normal or overexpressed) were detected with immunohistochemistry (IHC). MGMTp methylation status was assessed at the Department of Clinical Pathology, Medical Service, Region Skåne, via methylation-specific PCR (polymerase chain reaction). An MGMT promoter methylation level >10% was considered positive for methylation. IDH 1 and 2 status (IDH-mut and IDH-wt) was analyzed either by IHC or quantitative PCR, with IDH-mut defined as having either IDH1 or IDH2 mutation (in this cohort, all IDH-mutated cases were IDH1 mutated, none were IDH2 mutated). ATRX-mut was defined as the complete loss of ATRX expression. In the final cohort, there were no patients who exhibited partially intact ATRX expression.

### 2.5. Statistical Analysis

Statistical analysis was performed with SPSS^®^ v. 26.0 (IBM Corp., New York, NY, USA; formerly SPSS Inc., Chicago, IL, USA). Boxplots, skewness, kurtosis, and outliers were assessed and a Shapiro–Wilk’s test conducted to test normality in distribution. When testing for normality and unequal sample groups, mixed distributions favored the Mann–Whitney U test (MW-U) to quantify the APTw% signal.

The mean, median, and maximum signal within abnormally appearing tissue on Gd enhanced T1-MPRAGE and FLAIR maps were examined with the MW-U test. The HGGs were assessed for differences in APTw% signal stratified over wild-type or mutated ATRX and IDH, MGMTp with and without methylation, Ki-67 index above the level of 10% and p53 expression. A combined logistic regression model was constructed for HGG with normal p53 vs. overexpressed p53. A subgroup analysis of the same parameters was performed in the 36 IDH-wt HGG defined as GBM (glioblastoma grade 4), according to the WHO CNS5/2021 classification. Receiver operating characteristic (ROC) curves were assessed and area under the curve (AUC), sensitivity, and specificity were calculated, with a significance level set at *p*-value < 0.05 [36]. A Hanley–McNeil test was performed between mean and median APTw (%) ROC curves for p53 and MGMTp methylation status [61]. Statistical analysis was performed with SPSS^®^ v. 29.0 (IBM Corp., New York, NY, USA; formerly SPSS Inc., Chicago, IL, USA).

## 3. Results

Patient demographics and clinical characteristics are listed in Table 1. Mean age at APTw MRI examination differed significantly between IDH-wt (*n* = 36) and IDH-mut (all six being IDH 1 mutated), *p* = 0.001, as well as between ATRX-wt (*n* = 36) and ATRX-mut (*n* = 6), *p* = 0.007, MW-U test (Table 1). High-grade gliomas stratified over age at the date of the first obtained APTw MRI examination were not found to be statistically significant for MGMTp, *p* = 0.7; Ki-67 index, *p* = 0.8; and p53 status, *p* = 0.4 (Table 1 and Table S5).

The molecular status and most frequent corresponding anatomical lesion locations are shown in Table 1 and Table 2. The most frequent locations for HGGs were frontal lobes (*n* = 12), temporal lobes (*n* = 8), and parietal lobes (*n* = 6). Eleven HGGs occupied at least two lobes with a predilection for frontal and parietal (*n* = 5) distribution; 22 of the 42 HGGs were in the left hemisphere and 17 were in the right hemisphere, not counting the three cases that had crossed the midline via the corpus callosum. Stratified over molecular status and location, as shown in Table 2, tumors with normal p53 expression were frontal sin (five instances) and dx (five instances), temporal sin (four instances) and dx (four instances), and parietal sin (three) and dx (six). HGGs having non-methylated MGMTp, in total 21 cases, favored the following locations: parietal sin (six instances) and dx (two instances), and frontal sin (five instances) and dx (one instance). The HGGs with methylated MGMTp, in total 15 cases, had mainly a frontal location: frontal sin (four instances) and dx (six instances), parietal dx (six instances), and temporal sin (four instances) and dx (three instances). In 4/42 subjects, MGMT status was not accessible or unknown.

**Figure 2:**
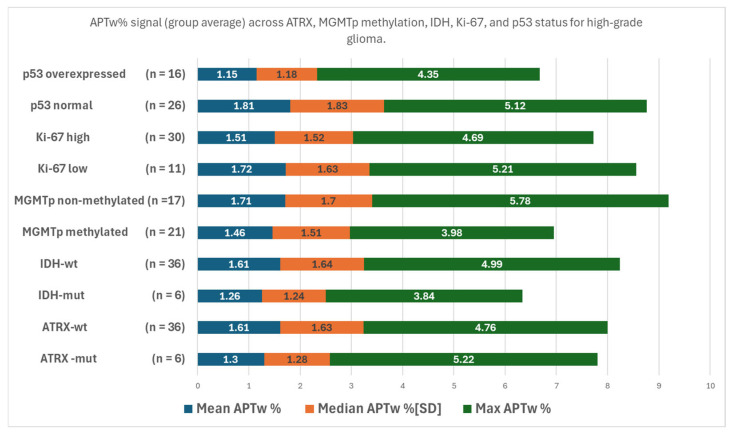
Composite graph showing the mean, median, and max APTw% signal for the different expressions of molecular markers (ATRX-mut, ATRX-wt, IDH-mut, IDH-wt, MGMTp non-methylated, MGMTp methylated, Ki-67 low, Ki-67 high, and normal or overexpressed p53). HGGs stratified over molecular status were not found to be statistically significant for mean, median, and max APTw% signal: ATRX: *p*-values = 0.3, 0.3, and 0.3; IDH: *p*-values = 0.3, 0.2, and 0.5; and Ki-67 index: *p*-values = 0.1, 0.3, and 0.6, respectively (Table S5).

HGGs with non-methylated MGMTp exhibited the highest max APTw% signal at 5.78, closely followed by HGGs with normally expressed p53. The HGGs with methylated MGMTp showed less mean-, median and max APTw% signal vs the HGGs that were non-methylated (Figure 2). HGGs stratified over molecular status; IDH, ATRX, Ki-67 exhibited differences in mean, median, and max APTw% signal but the trends were not statistically significant (Figure 2).

**Figure 3:**
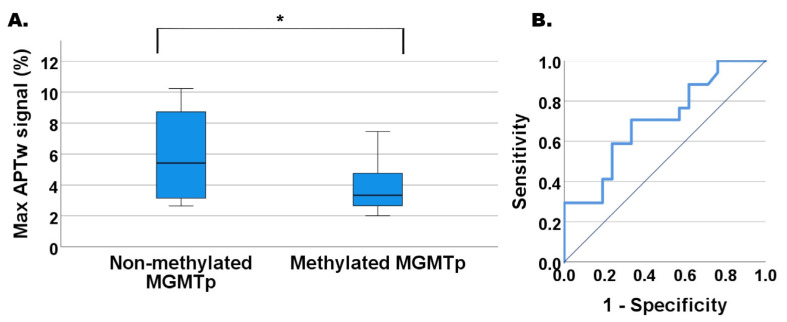
Max APTw% signal for HGG with MGMTp methylation vs. without MGMTp methylation. (**A**) Boxplot. Methylated MGMTp has a reduced max APTw% signal compared to the non-methylated MGMTp. (**B**) ROC curve. AUC = 0.702, *p*-value = 0.035 for ROC curve. Asymp. 95% CI 0.534–0.869. Max APTw% signal cutoff of 4.28% yields 70.6% sensitivity and 66.7% specificity. Non-methylated *n* = 17 and methylated *n* = 21 (MGMTp analysis was not accessible in four cases). * Denotes statistical significance. Hanley–McNeil test yielded an AUC difference of 0.084 with a standard error of 0.1095, z = 0.7668, and *p*-value 0.44 between max APTw (%) for MGMTp methylation/non-methylated mean APTw (%) for p53 overexpression/normal ROC curve metrics. This indicates no statistically significant differences in ROC performance between the two groups.

The mean, median, and max APTw% signal intensities were evaluated for HGG stratified over MGMTp methylation status as exemplified in Figure 3 for max APTw% signal (*p* = 0.035).

HGGs with non-methylated MGMTp exhibited higher max APTw% signal vs. methylated MGMTp (5.78% vs. 3.98% signal). The ROC analysis of max APTw% signal for MGMTp methylation status is shown in Figure 3B. The MW-U test *p*-value = 0.035 and cutoff value of max APTw% signal = 4.28% (*n* = 17 non-methylated MGMTp; *n* = 21 methylated MGMTp) gave a 70.6% sensitivity and 66.7% specificity with an AUC of 0.702 (*p*-value = 0.035). Mean APTw (1.71% vs. 1.46%) and median APTw (1.70% vs. 1.51%) signals for non-methylated MGMT showed only trends of being significantly larger than methylated MGMTp, *p* > 0.05.

When analyzing the cohort of 36 HGG individuals defined as having glioblastoma (GBM) according to WHO CNS5/2021, with IDH-wt per definition, the difference of max APTw, mean, and median APTw between MGMT methylated and non-methylated tumors was found to be non-significant (*p* = 0.078, 0.885; 0.601) (Tables S1 and S2).

**Figure 4:**
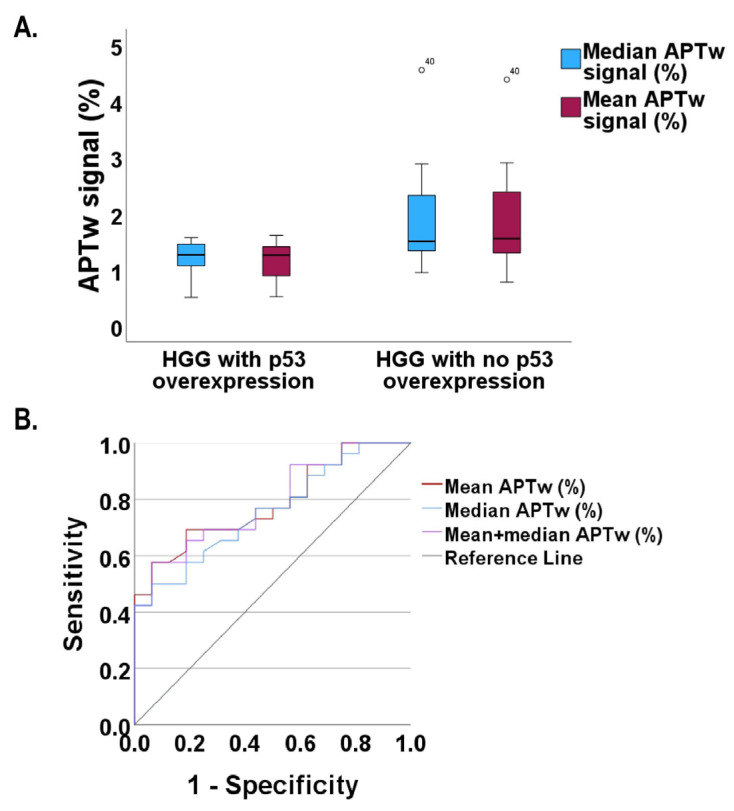
APTw% signals for high-grade glioma with and without overexpressed p53. (**A**) Clustered boxplots showing the distribution of mean APTw% signal and median APTw% signal. APTw% signal was reduced in HGG with an overexpressed p53. (**B**) ROC curves. AUC = 0.786/0.757/0.788 for mean/median/mean + median APTw% signals, with MW-U test *p*-values = 0.002/0.006/0.002, respectively. See Table 3 for corresponding sensitivities, specificities, and 95% CI. HGGs with normal p53, *n* = 26, and HGG with overexpressed p53, *n* = 16. Hanley–McNeil test yielded an AUC difference of 0.029 with a standard error of 0.1016, z = 0.2855, and *p*-value 0.78 between mean and median APTw (%) ROC curve metrics. This indicates no statistically significant differences in ROC performance between the two groups. Number 40 and circles pertain to patient 40 being an outlier for mean and median APTw% signals.

Mean and median APTw% signals showed potential when differentiating between p53 expression status (Figure 2 and Figure 4 and Table 3 and Table S5). The mean APTw% signal for p53 normal vs. overexpressed HGGs was 1.81% vs. 1.15% (*p* = 0.002) and the median APTw% signal was 1.83% vs. 1.18% (*p* = 0.006), respectively. This relation of mean/median APTw% signals also remained significant in the 36 GBMs according to WHO CNS5/2021, at 1.86%/1.87% vs. 1.11%/1.16, *p* = 0.02/0.08, respectively (Tables S2 and S4). However, the max APTw% signal differentiation was found to be not statistically significant (Table S2) in any case. ROC analysis (Figure 4B) and the predictive capacity of mean and median APTw% signals for p53 status in the 42 HGGs was 76.9% and 66.2% in terms of sensitivity and specificity with a probability cutoff of 0.57 (*p* = 0.002) (Table 3). The ROC in HGG yielded a cutoff/sensitivity/specificity of 1.25%/76.9%/50% for the mean APTw% signal and 1.33%/76.9%/66.2% for the median APTw% signal (Table 3). The combined logistic regression model for mean + median APTw% signal showed 0.57/76.9%/66.2% for probabilistic cutoff/sensitivity/specificity (Table 3). AUC for the combined model (0.788) was higher than for the mean, 0.786, and median, 0.757, APTw% signal (Table 3).

## 4. Discussion

The primary aim of this study was to assess the capability of APTw imaging in differentiating high-grade glial tumors based on their p53/ATRX/IDH/Ki-67 index/MGMTp methylation status. We showed that APTw measurements obtained from the manual segmentation of enhancing solid tumor (Figure 1) have the potential to non-invasively differentiate HGGs based on their p53 status. In the present study, the non-methylated MGMTp HGG exhibited a significantly higher max APTw% signal vs. methylated tumors in the 42 HGG cases according to WHO 2016 classification including the six IDH-mutated cases, but this finding did not reach significance when re-analyzed to include only the 36 IDHwt glioblastoma patients according to the WHO CNS5/2021 classification (Table S2). HGGs with normal p53 expression showed significantly higher mean, median, and mean + median APTw% signals vs. HGGs with overexpressed p53, both in the GBM cohort according to WHO 2016 classification and the present WHO CNS5/2021 (Tables S2 and S4).

To our knowledge, no previous study has explored p53/ATRX/IDH/Ki-67 index/MGMTp methylation status within the same cohort of HGG patients, in relation to APTw. There are conflicting results in the literature with respect to molecular biomarkers and APTw% signal, recently summarized in a meta-analysis by Jiang [9]. Some studies have reported significant differences in APTw% signal between HGG with and without MGMTp methylation [52] and others have reported no differences between IDH-wt HGG stratified across MGMTp methylation status and p53 status [62], or MGMTp methylation status in low- and high-grade gliomas [48]. Similarly, for different types of HGGs, including both anaplastic astrocytoma and GBM, there were no significant differences across MGMTp status [54].

As additionally highlighted by Jiang [9], earlier studies have used a mixed cohort of gliomas including WHO Grades II–IV or III–IV, and investigated either MGMT and IDH status alone or together [48,53,54]. Of the studies mentioned, one used the 2007 WHO classification [53] and another had a limited number of patients with Grade IV glioma (18 patients, all males) [52].

In our study, we could not establish a relationship between Ki-67 index, IDH1/2, and ATRX status and mean, median, and max APTw% signals (Table S5). Neither could we identify significant differences in APTw% signal in relation to MGMT status for the 36 cases of GBM according to WHO CNS5/2021 (Table S2).

However, we observed that when analyzing the whole cohort of 42 HGG, also including the six cases of IDH-mutated tumors, the non-methylated MGMTp HGGs had significantly higher max APTw% signals compared to the methylated MGMTp HGGs within the entire segmented enhancing solid part of the lesion (Figure 3A). Similarly to our findings in Figure 3B, Jiang et al. [52] showed, in a smaller cohort, a finding where the 90th percentile APTw% signal had the best discriminatory capacity with AUC = 0.856 for eight MGMTp non-methylated vs. eleven methylated GBMs [52]. However, they did not account for ATRX/IDH status or Ki-67 index, which may impact the interpretation of the results due to inferred bias.

Alkylating agents such as temozolomide impact DNA at multiple sites with the O^6^-group in guanine receiving most of the damage through alkylation [63]. The MGMT gene counteracts the alkylation through the MGMT protein while the methylation of the MGMT promoter silences the gene and, by proxy, induces reduced repair of DNA [26,63]. However, different molecular fingerprints can impact survival [64] in either direction as the complexity of glioma pathogenicity and genetics increases. For example, it was shown that despite a BRAF-amplified glioma harboring IDH1/2 and ATRX mutations, known for a better prognosis, a reduced survival was observed vs. V600E mutation v-Raf murine sarcoma viral oncogene homolog B1 BRAF^v600E^ [64].

As MGMTp methylation has been shown to impact survival regardless of the use of alkylating agents [26], finding noninvasive methods for stratifying patients with HGG in less and more favorable survival groups prior to surgical resection or biopsy has become even more important. In the preoperative setting, information regarding MGMTp methylation status could potentially aid in deciding the best surgical approach (biopsy vs. resection) for gliomas located within or near eloquent areas. Non-invasive information regarding MGMTp methylation status is, at the very least, of importance for brainstem lesions where biopsy is associated with significant risks. However, further studies are needed and Wang et al. [64] also showed that there is a need for future imaging studies on patients with several simultaneous histologic mutations due to the down- and upstream effects of concurrent mutations [6,64].

APTw imaging could distinguish HGGs and, specifically, grade 4 GBMs according to WHO CNS5/2021 with normal p53 from those with overexpressed p53. A previous study also used APTw imaging to predict p53 status in rectal adenocarcinoma with a higher mean APTw% signal in tumors with overexpressed p53 status [65]. The ability to predict p53 status is of importance, especially in gliomas, since it correlates with the pathological grade, with higher-grade gliomas exhibiting a higher frequency of p53 immuno-positivity/overexpression vs. low-grade gliomas [66]. More recent research has shown that the overexpressed and often mutated p53 protein also has negative effects on normally expressed p53 through, among others, gain-of-function (GOF) mutations which drive tumorigenesis as other oncogenes [17,67,68,69,70]. Several other studies have shown that the higher frequency of p53 overexpression in breast cancer also correlates well with mutations of the TP53 gene. This is not solely attributed to overexpressed p53 being more stable than the normal p53 [71], but also to the above-mentioned GOF mutations [17,67,68,69,70], which seem to impart a survival benefit for the transformed cell [67,68,69,70]. The higher degree of mutations in the TP53 gene is associated with GBM cell lines with a higher resistance to cisplatin treatment and the cells with mutated and inactivated TP53 and a deregulated p53 pathway exhibit a higher degree of invasiveness, less apoptosis, and more proliferation than their wild-type TP53 counterparts [18]. However, the function of TP53 wild type may also be influenced by additional factors, making these tumors relevant in the future, for other types of specific therapeutic strategies, such as targeting the bromodomain of the chromatin regulator bromodomain-containing protein 8 (BRD8) [22].

Shifeng et al. [72] examined endometrial carcinoma and p53 status. They found that the APTw% signal was higher in the overexpressed p53 group vs. the group with normal p53 expression, albeit with lower ADC [72]. AUCs for APTw, ADC, and the combined prediction model with APTw + R2* + ADC values were 0.739, 0.718, and 0.820, respectively, all with significant *p*-values < 0.05 (70). In our study, the AUC was higher in the logistic regression model vs. the single predictors mean and median APTw% signal (Table 3). The high expression of mutant p53 was found to correlate with temporal lobe tumor involvement in a cohort of low-grade glioma patients [73], while in our cohort we observed overexpressed p53 domination at frontal sin (five instances) and dx (three instances) locations (Table 2).

Some recent studies used conventional MR imaging with or without contrast (T1w, T2w, and T2w FLAIR) [74], T2w imaging [75], and conventional MR imaging concomitantly with susceptibility-weighted, diffusion-weighted, and diffusion tensor imaging and arterial spin labeling [76] to predict p53 status in different brain tumors such as diffuse intrinsic pontine glioma, low-grade glioma, and GBM [74,75,76]. These studies demonstrate similar AUC, specificity, and sensitivity to predict p53 as our study. However, contradictory to our study, these studies did not evaluate all of the different conventional molecular analyses. In addition, they did not evaluate the APTw imaging as a potential imaging biomarker in the evaluation of p53 status.

## 5. Limitations

There are a few limitations to our study.

An essential challenge was the change in definition in 2021 of grade 4 glioblastoma to only include IDH-wt, a situation shared with other publications during this period, no longer giving significance to the max APTw% signal difference between MGMTp non-methylated and methylated GBM cases. However, we do assume a clinical value in the preoperative setting of the max APTw% signal in differentiation between MGMTp non-methylated vs. MGMT methylated cases of HGG when IDH status is still unknown, since this may influence the strategy in surgical approach due to a different prognosis between MGMTp non-methylated and MGMT methylated cases, both among IDH-mutated and IDH-wt HGGs. Irrespective of IDH status, an indication of the MGMT status as assessed by APTw MRI is of clear value for preoperative planning and the determination of the surgical strategy in a contrast-enhancing suspected glioma.

Even if the Mann–Whitney U test is robust as an analysis for a small number of patients and groups of unequal sizes, this study would benefit from a larger cohort. Treatment effects, which may influence the APTw% signal, were not adjusted for. Manual tumor segmentation is a cumbersome method, and this study might have benefited from an automated machine learning approach for the whole tumor segmentation.

Even though we do not account for some mutations such as BRAF, as they are not performed routinely at our clinic, our findings strengthen previous studies on APTw imaging for predicting molecular status [9,48,53,54]. Our study does not accord for the phosphorylation, acetylation, or methylation of the TP53 gene, epigenetic modifications that may impact the stability of the protein product of the gene TP53 [17].

A total of 10 out of 42 patients were diagnosed with stereotactic biopsy while the remaining 32 patients had undergone surgical resection. Although only a small subset of the patients had a biopsy (24% of the total), these may harbor additional mutations due to the metabolic tumor heterogeneity, causing a potential sampling bias.

Metrics such as ADC and cerebral blood flow (CBF)/cerebral blood volume (CBV%) were out of scope for this work; however, additional parameters would, from a diagnostic perspective, be of interest since an improved accuracy using a combined logistic regression model might have been obtained.

There is an important technical limitation, namely the use of a relatively short saturation time for the APTw imaging, resulting in the contrast to noise being lower compared to what can be expected with a longer saturation time. The short saturation time together with a B1 of 2μT enhances the contrast from vascular proteins [77]. Thus, the increase in tumors may be in part due to a large blood-based component. This study was completed before consensus recommendations for APTw MRI were established [78], and a sequence using the recommended settings [79] became available. However, the results are still clinically relevant.

## 6. Conclusions

This study reports quantitative APTw MRI results for simultaneously differentiating ATRX/MGMTp methylation/Ki-67 index/IDH status and p53 expression in WHO grade 4 high-grade glioma (thirty-six IDH wild-type glioblastoma and six IDH-mutant astrocytoma) considering both WHO 2016 and WHO CNS5/2021 classifications. The results indicate that the mean + median APTw% signal can distinguish p53 normal versus p53-overexpressed high-grade glioma. Non-methylated MGMTp HGGs have a higher max APTw% signal compared to HGGs with MGMTp methylated expression. We conclude that APTw imaging has the potential to serve as an additional clinical noninvasive tool to aid in future preoperative decision making and precision medicine.

## Figures and Tables

**Table 1:** Demographics, mutation status, and lesion location for a total of 42 high-grade glioma (HGG) patients.

Histological or Demographic Features		Count
Biological sex	Female	12
	Male	30
ATXR wild type	No	6
	Yes	36
IDH wild type	No	6
	Yes	36
Ki-67 index > 10% (%)	No	11
	Yes	30
MGMT promoter methylation **	No	17
	Yes	21
p53 wild type	No	16
	Yes	26
Age at APTw MRI exam (years)	Overall (y)	56
	Male vs. female (y)	56 vs. 58
	MGMTp meth vs. non-meth (y)	58 vs. 56
	ATRX-wt vs. ATRX-mut (y)	57 vs. 49 *
	IDH-wt vs. IDH-mut (y)	59 vs. 38 *
	Ki-67-low vs. Ki-67-high (y)	56 vs. 56
	P53-wt vs. p53-overexpressed	58 vs. 51
Lesion location	Frontal dx	4
	Frontal sin	8
	Fronto–parietal dx	1
	Fronto–parietal sin	1
	Fronto–insulo–parietal dx	1
	Fronto–insulo–parietal sin	1
	Fronto–insulo–temporal dx	1
	Fronto–tempero-–parietal dx	1
	Midline CC	3
	Occipital sin	2
	Parietal dx	4
	Parietal sin	2
	Parieto–occipital dx	1
	Parieto–occipital sin	1
	Temporal dx	2
	Temporal sin	6
	Tempero–parietal dx	1
	Tempero–parietal sin	1
	Tempero–occipital dx	1

* *p*-value < 0.05 and denotes a statistically significant difference. ** Missing cases in total: MGMT promoter methylation (*n* = 4) and Ki-67 index (*n* = 1).

**Table 2:** Spatial distribution ^#^ of overexpressed p53, normally expressed p53, non-methylated MGMTp, and methylated MGMTp in different brain regions.

Anatomical Lesion Location	Frontal		Temporal		Parietal	
High-grade glioma and molecular characteristic	Sin	Dx	Sin	Dx	Sin	Dx
Overexpressed p53	5	3	-	-	-	-
Normally expressed p53	5	5	4	4	3	6
Non-methylated MGMTp	5	1	-	-	6	2
Methylated MGMTp	4	6	4	3	0	6

^#^ The frequency is presented for each type in frontal, temporal, and parietal regions, further categorized by hemisphere with ‘sin’ representing the left brain hemisphere and ‘dx’ representing the right brain hemisphere.

**Table 3:** ROC analysis showing utilization of mean and median APTw% signals and their combination through logistic regression for distinguishing p53 normally expressed in HGGs vs. p53 overexpressed in HGGs.

	Area Under the Curve (AUC)	*p*-Value	95% Confidence Interval		Cutoff/Sensitivity/Specificity
			Lower Bound	Upper Bound	
Mean APTw	0.786	0.002	0.65	0.92	1.25%/76.9%/50.0%
Median APTw	0.757	0.006	0.61	0.90	1.33%/76.9%/66.2%
Mean + median APTw	0.788	0.002	0.65	0.92	0.57 */76.9%/66.2%

* Probabilistic value provided by the logistic regression model and not APTw% signal value. HGGs with normal p53, *n* = 26, and HGGs with overexpressed p53, *n* = 16.

## Data Availability

All relevant data are within the paper. However, the in vivo data cannot be made publicly available, as this would violate Swedish law. The research was performed under an IRB approval and required the research subject to sign informed consent. According to Swedish law applicable to this study, the scope of the consent must be specific (Personal Data Act 1998:204; Swe. “Personuppgiftslagen”, http://rkrattsbaser.gov.se/sfst?bet=1998:204 (accessed on 24 October 1998)). Therefore, we are prohibited from sharing the data publicly for general research that was not described in the consent form. Data are available upon request from researchers who have ethical approval to Assoc Professor Markus Nilsson, at the Institution of Clinical Sciences, Department of Radiology and Deputy group leader for the MR physics group at the Department of Medical Radiation Physics Markus Nilsson, (Address: Dept. of Medical Radiation Physics, Skane University Hospital Lund, Barngatan 6, Lund 22185, Sweden; markus.nilsson@med.lu.se).

## Supplementary Materials

The following supporting information can be downloaded at: https://www.mdpi.com/article/10.3390/tomography11060064/s1, Table S1: Demographics, mutation status, for a total of 36 Glioblastoma patients after re-classification according to WHO CNS5 /2021 classification.; Table S2: APTw% signal (group average) across ATRX-, MGMTp methylation-, IDH- and Ki-67-status, p53 status (after re-classification according to the WHO CNS 5/2021 classification for Glioblastoma (n = 36 IDH-wt))Table S3: Spatial distribution# of overexpressed p53, normal p53, non-methylated MGMTp, and methylated MGMTp in different brain regions in 36 Glioblastoma patients after re-classification according to WHO CNS /2021 classification.; Table S4:ROC analysis of mean- and median- APTw% signal and their combination through logistic regression for distinguishing p53 normal expressed Glioblastoma vs p53 overexpressed in 36 Glioblastoma patients after re-classification according to WHO CNS /2021 classification; Table S5: APTw% signal (group average) across ATRX-, MGMTp methylation-, IDH- and Ki-67-status, p53 status for high grade glioma; Table S6: The molecular and genetic profiles for each patient along with type of surgery for obtaining tissue that was sent for histopathological analysis and histological diagnosis according to both the WHO 2016 CNS tumor classification and the most recent one from 2021.

## Abbreviations

ADC	apparent diffusion coefficient
APT	amide proton transfer
APTw	amide proton transfer weighted
ATRX	alpha-thalassemia/mental retardation, X-linked
AUC	area under the curve
BRAF	B-raf proto oncogene
CBF	cerebral blood flow
CBV	cerebral blood volume
CDKN2A/B	cyclin-dependent kinase inhibitor 2A or 2B
CEST	chemical exchange saturation transfer
DSC PW	dynamic susceptibility contrast perfusion weighted imaging
EGFR	epidermal growth factor receptor
FLAIR	fluid attenuation inversion recovery
GBM	Glioblastoma WHO Grade IV/4
Gd	gadolinium
GOF	gain of function mutation
GTR	gross total resection
H3K27M	lysine-to-methionine substitution at position 27 (K27M) in histone H3
HGG	High-grade glioma
IDH	isocitrate dehydrogenase
IHC	immunohistochemistry
Ki-67 index	antigen Kiel 67 proliferation index
LGG	Low-grade glioma
MGMT	O6-methylguanine-DNA methyltransferase
MGMTp	MGMT promoter
MLPA	multiplex ligation-dependent probe amplification
MPRAGE	magnetization prepared rapid acquisition gradient echo
mut	mutated
MW-U	Mann–Whitney U test
OS	overall survival
p53	tumor protein 53
PCR	polymerase chain reaction
PFS	progression-free survival
PTEN	Phosphatase and Tensin homolog gene
ROC	receiver operating characteristic
T1c	T1-weighted imaging with Gd enhancement
TERT	telomerase reverse transcriptase
TP53	TP 53 gene
WHO	World Health Organization
WHO CNS5/2021	WHO classification of CNS tumors 2021
wt	wild type
Dx	dexter (Latin for ‘right’)
Sin	sinister (Latin for ‘left’)

## References

[1] Fan H.C., Chen C.M., Chi C.S., Tsai J.D., Chiang K.L., Chang Y.K., Lin S.Z., Harn H.J. (2019). Targeting Telomerase and ATRX/DAXX Inducing Tumor Senescence and Apoptosis in the Malignant Glioma. Int. J. Mol. Sci..

[2] Thakkar J.P., Dolecek T.A., Horbinski C., Ostrom Q.T., Lightner D.D., Barnholtz-Sloan J.S., Villano J.L. (2014). Epidemiologic and molecular prognostic review of glioblastoma. Cancer Epidemiol. Biomarkers Prev..

[3] Ohka F., Natsume A., Wakabayashi T. (2012). Current trends in targeted therapies for glioblastoma multiforme. Neurol. Res. Int..

[4] Wang W., Shi G., Ma B., Hao X., Dong X., Zhang B. (2017). Chemotherapy for Adults with Malignant Glioma: A Systematic Review and Network Meta-Analysis. Turk. Neurosurg..

[5] Louis D.N., Perry A., Wesseling P., Brat D.J., Cree I.A., Figarella-Branger D., Hawkins C., Ng H.K., Pfister S.M., Reifenberger G. (2021). The 2021 WHO Classification of Tumors of the Central Nervous System: A summary. Neuro Oncol..

[6] Karsy M., Guan J., Cohen A.L., Jensen R.L., Colman H. (2017). New Molecular Considerations for Glioma: IDH, ATRX, BRAF, TERT, H3 K27M. Curr. Neurol. Neurosci. Rep..

[7] Louis D.N., Perry A., Reifenberger G., von Deimling A., Figarella-Branger D., Cavenee W.K., Ohgaki H., Wiestler O.D., Kleihues P., Ellison D.W. (2016). The 2016 World Health Organization Classification of Tumors of the Central Nervous System: A summary. Acta Neuropathol..

[8] van den Bent M.J., Weller M., Wen P.Y., Kros J.M., Aldape K., Chang S. (2017). A clinical perspective on the 2016 WHO brain tumor classification and routine molecular diagnostics. Neuro Oncol..

[9] Jiang S., Wen Z., Ahn S.S., Cai K., Paech D., Eberhart C.G., Zhou J. (2023). Applications of chemical exchange saturation transfer magnetic resonance imaging in identifying genetic markers in gliomas. NMR Biomed..

[10] Ichimura K., Pearson D.M., Kocialkowski S., Backlund L.M., Chan R., Jones D.T., Collins V.P. (2009). IDH1 mutations are present in the majority of common adult gliomas but rare in primary glioblastomas. Neuro Oncol..

[11] Ren Y., Zhang X., Rui W., Pang H., Qiu T., Wang J., Xie Q., Jin T., Zhang H., Chen H. (2019). Noninvasive Prediction of IDH1 Mutation and ATRX Expression Loss in Low-Grade Gliomas Using Multiparametric MR Radiomic Features. J. Magn. Reson. Imaging.

[12] Wang Z.L., Zhang C.B., Cai J.Q., Li Q.B., Wang Z., Jiang T. (2015). Integrated analysis of genome-wide DNA methylation, gene expression and protein expression profiles in molecular subtypes of WHO II-IV gliomas. J. Exp. Clin. Cancer Res..

[13] Doll S., Urisman A., Oses-Prieto J.A., Arnott D., Burlingame A.L. (2017). Quantitative Proteomics Reveals Fundamental Regulatory Differences in Oncogenic HRAS and Isocitrate Dehydrogenase (IDH1) Driven Astrocytoma. Mol. Cell Proteomics.

[14] Nandakumar P., Mansouri A., Das S. (2017). The Role of ATRX in Glioma Biology. Front. Oncol..

[15] Leeper H.E., Caron A.A., Decker P.A., Jenkins R.B., Lachance D.H., Giannini C. (2015). IDH mutation, 1p19q codeletion and ATRX loss in WHO grade II gliomas. Oncotarget.

[16] Kannan K., Inagaki A., Silber J., Gorovets D., Zhang J., Kastenhuber E.R., Heguy A., Petrini J.H., Chan T.A., Huse J.T. (2012). Whole-exome sequencing identifies ATRX mutation as a key molecular determinant in lower-grade glioma. Oncotarget.

[17] Sabapathy K., Lane D.P. (2019). Understanding p53 functions through p53 antibodies. J. Mol. Cell Biol..

[18] Zhang Y., Dube C., Gibert M., Cruickshanks N., Wang B., Coughlan M., Yang Y., Setiady I., Deveau C., Saoud K. (2018). The p53 Pathway in Glioblastoma. Cancers.

[19] Niskanen E., Blomqvist C., Franssila K., Hietanen P., Wasenius V.M. (1997). Predictive value of c-erbB-2, p53, cathepsin-D and histology of the primary tumour in metastatic breast cancer. Br. J. Cancer.

[20] Kandioler-Eckersberger D., Ludwig C., Rudas M., Kappel S., Janschek E., Wenzel C., Schlagbauer-Wadl H., Mittlbock M., Gnant M., Steger G. (2000). TP53 mutation and p53 overexpression for prediction of response to neoadjuvant treatment in breast cancer patients. Clin. Cancer Res..

[21] Lowe S.W., Schmitt E.M., Smith S.W., Osborne B.A., Jacks T. (1993). p53 is required for radiation-induced apoptosis in mouse thymocytes. Nature.

[22] Sun X., Klingbeil O., Lu B., Wu C., Ballon C., Ouyang M., Wu X.S., Jin Y., Hwangbo Y., Huang Y.H. (2023). BRD8 maintains glioblastoma by epigenetic reprogramming of the p53 network. Nature.

[23] Scholzen T., Gerdes J. (2000). The Ki-67 protein: From the known and the unknown. J. Cell Physiol..

[24] Johannessen A.L., Torp S.H. (2006). The clinical value of Ki-67/MIB-1 labeling index in human astrocytomas. Pathol. Oncol. Res..

[25] Togao O., Yoshiura T., Keupp J., Hiwatashi A., Yamashita K., Kikuchi K., Suzuki Y., Suzuki S.O., Iwaki T., Hata N. (2014). Amide proton transfer imaging of adult diffuse gliomas: Correlation with histopathological grades. Neuro Oncol..

[26] Hegi M.E., Diserens A.C., Gorlia T., Hamou M.F., de Tribolet N., Weller M., Kros J.M., Hainfellner J.A., Mason W., Mariani L. (2005). MGMT gene silencing and benefit from temozolomide in glioblastoma. N. Engl. J. Med..

[27] Weller M., Felsberg J., Hartmann C., Berger H., Steinbach J.P., Schramm J., Westphal M., Schackert G., Simon M., Tonn J.C. (2009). Molecular predictors of progression-free and overall survival in patients with newly diagnosed glioblastoma: A prospective translational study of the German Glioma Network. J. Clin. Oncol..

[28] McAleenan A., Kelly C., Spiga F., Kernohan A., Cheng H.Y., Dawson S., Schmidt L., Robinson T., Brandner S., Faulkner C.L. (2021). Prognostic value of test(s) for O6-methylguanine-DNA methyltransferase (MGMT) promoter methylation for predicting overall survival in people with glioblastoma treated with temozolomide. Cochrane Database Syst. Rev..

[29] Wick W., Platten M., Meisner C., Felsberg J., Tabatabai G., Simon M., Nikkhah G., Papsdorf K., Steinbach J.P., Sabel M. (2012). Temozolomide chemotherapy alone versus radiotherapy alone for malignant astrocytoma in the elderly: The NOA-08 randomised, phase 3 trial. Lancet Oncol..

[30] Liu X.Y., Gerges N., Korshunov A., Sabha N., Khuong-Quang D.A., Fontebasso A.M., Fleming A., Hadjadj D., Schwartzentruber J., Majewski J. (2012). Frequent ATRX mutations and loss of expression in adult diffuse astrocytic tumors carrying IDH1/IDH2 and TP53 mutations. Acta Neuropathol..

[31] Wen P.Y., Packer R.J. (2021). The 2021 WHO Classification of Tumors of the Central Nervous System: Clinical implications. Neuro Oncol..

[32] Wu L., Chai R., Lin Z., Wu R., Yao D., Jiang T., Wang Q. (2023). Evolution-driven crosstalk between glioblastoma and the tumor microenvironment. Cancer Biol. Med..

[33] Wu L., Wu W., Zhang J., Zhao Z., Li L., Zhu M., Wu M., Wu F., Zhou F., Du Y. (2022). Natural Coevolution of Tumor and Immunoenvironment in Glioblastoma. Cancer Discov..

[34] Goffart N., Lombard A., Lallemand F., Kroonen J., Nassen J., Di Valentin E., Berendsen S., Dedobbeleer M., Willems E., Robe P. (2017). CXCL12 mediates glioblastoma resistance to radiotherapy in the subventricular zone. Neuro Oncol..

[35] Rubenich D.S., de Souza P.O., Omizzollo N., Aubin M.R., Basso P.J., Silva L.M., da Silva E.M., Teixeira F.C., Gentil G.F.S., Domagalski J.L. (2023). Tumor-neutrophil crosstalk promotes in vitro and in vivo glioblastoma progression. Front. Immunol..

[36] Rao J.U., Coman D., Walsh J.J., Ali M.M., Huang Y., Hyder F. (2017). Temozolomide arrests glioma growth and normalizes intratumoral extracellular pH. Sci. Rep..

[37] Estrella V., Chen T., Lloyd M., Wojtkowiak J., Cornnell H.H., Ibrahim-Hashim A., Bailey K., Balagurunathan Y., Rothberg J.M., Sloane B.F. (2013). Acidity generated by the tumor microenvironment drives local invasion. Cancer Res..

[38] Coman D., Huang Y., Rao J.U., De Feyter H.M., Rothman D.L., Juchem C., Hyder F. (2016). Imaging the intratumoral-peritumoral extracellular pH gradient of gliomas. NMR Biomed..

[39] Zhou J., Payen J.F., Wilson D.A., Traystman R.J., van Zijl P.C. (2003). Using the amide proton signals of intracellular proteins and peptides to detect pH effects in MRI. Nat. Med..

[40] Knutsson L., Xu J., Ahlgren A., van Zijl P.C.M. (2018). CEST, ASL, and magnetization transfer contrast: How similar pulse sequences detect different phenomena. Magn. Reson. Med..

[41] Yamauchi T., Kitai R., Kodera T., Arishima H., Matsuda K., Isozaki M., Ishida S., Matta Y., Kanamoto M., Kimura H. (2022). Comparison of amide proton transfer imaging with perfusion imaging of using arterial spin-labeling for evidence of tumor invasion in glioblastoma. Interdiscip. Neurosurg..

[42] Zhou J., Heo H.Y., Knutsson L., van Zijl P.C.M., Jiang S. (2019). APT-weighted MRI: Techniques, current neuro applications, and challenging issues. J. Magn. Reson. Imaging.

[43] Song Q., Zhang C., Chen X., Cheng Y. (2020). Comparing amide proton transfer imaging with dynamic susceptibility contrast-enhanced perfusion in predicting histological grades of gliomas: A meta-analysis. Acta Radiol..

[44] Zhou J., Tryggestad E., Wen Z., Lal B., Zhou T., Grossman R., Wang S., Yan K., Fu D.X., Ford E. (2011). Differentiation between glioma and radiation necrosis using molecular magnetic resonance imaging of endogenous proteins and peptides. Nat. Med..

[45] Ma B., Blakeley J.O., Hong X., Zhang H., Jiang S., Blair L., Zhang Y., Heo H.Y., Zhang M., van Zijl P.C. (2016). Applying amide proton transfer-weighted MRI to distinguish pseudoprogression from true progression in malignant gliomas. J. Magn. Reson. Imaging.

[46] Jiang S., Eberhart C.G., Lim M., Heo H.-Y., Zhang Y., Blair L., Wen Z., Holdhoff M., Lin D., Huang P. (2019). Identifying Recurrent Malignant Glioma after Treatment Using Amide Proton Transfer-Weighted MR Imaging: A Validation Study with Image-Guided Stereotactic Biopsy. Clin. Cancer Res..

[47] Jiang S., Zou T., Eberhart C.G., Villalobos M.A.V., Heo H.Y., Zhang Y., Wang Y., Wang X., Yu H., Du Y. (2017). Predicting IDH mutation status in grade II gliomas using amide proton transfer-weighted (APTw) MRI. Magn. Reson. Med..

[48] Paech D., Windschuh J., Oberhollenzer J., Dreher C., Sahm F., Meissner J.E., Goerke S., Schuenke P., Zaiss M., Regnery S. (2018). Assessing the predictability of IDH mutation and MGMT methylation status in glioma patients using relaxation-compensated multipool CEST MRI at 7.0 T. Neuro Oncol..

[49] Yao J., Chakhoyan A., Nathanson D.A., Yong W.H., Salamon N., Raymond C., Mareninov S., Lai A., Nghiemphu P.L., Prins R.M. (2019). Metabolic characterization of human IDH mutant and wild type gliomas using simultaneous pH- and oxygen-sensitive molecular MRI. Neuro Oncol..

[50] Han Y., Wang W., Yang Y., Sun Y.Z., Xiao G., Tian Q., Zhang J., Cui G.B., Yan L.F. (2020). Amide Proton Transfer Imaging in Predicting Isocitrate Dehydrogenase 1 Mutation Status of Grade II/III Gliomas Based on Support Vector Machine. Front. Neurosci..

[51] Moon W.J., Choi J.W., Roh H.G., Lim S.D., Koh Y.C. (2012). Imaging parameters of high grade gliomas in relation to the MGMT promoter methylation status: The CT, diffusion tensor imaging, and perfusion MR imaging. Neuroradiology.

[52] Jiang S., Rui Q., Wang Y., Heo H.Y., Zou T., Yu H., Zhang Y., Wang X., Du Y., Wen X. (2018). Discriminating MGMT promoter methylation status in patients with glioblastoma employing amide proton transfer-weighted MRI metrics. Eur. Radiol..

[53] Su L., Gao P., Lin S., Wu B., Qin W., Lin Y., Xue J. (2018). Predicting O6-Methylguanine-DNA Methyltransferase Protein Expression in Primary Low- and High-Grade Gliomas Using Certain Qualitative Characteristics of Amide Proton Transfer-Weighted Magnetic Resonance Imaging. World Neurosurg..

[54] Joo B., Han K., Ahn S.S., Choi Y.S., Chang J.H., Kang S.G., Kim S.H., Zhou J., Lee S.K. (2019). Amide proton transfer imaging might predict survival and IDH mutation status in high-grade glioma. Eur. Radiol..

[55] Sotirios B., Demetriou E., Topriceanu C.C., Zakrzewska Z. (2020). The role of APT imaging in gliomas grading: A systematic review and meta-analysis. Eur. J. Radiol..

[56] Knutsson M., Salomonsson T., Durmo F., Johansson E.R., Seidemo A., Lätt J., Rydelius A., Kinhult S., Englund E., Bengzon J. (2025). Differentiation between glioblastoma and solitary brain metastases using perfusion and amide proton transfer weighted MRI. Front. Neurosci..

[57] Bryant R.G. (1996). The dynamics of water-protein interactions. Annu. Rev. Biophys. Biomol. Struct..

[58] Jenkinson M., Bannister P., Brady M., Smith S. (2002). Improved optimization for the robust and accurate linear registration and motion correction of brain images. Neuroimage.

[59] Smith S.M. (2002). Fast robust automated brain extraction. Hum. Brain Mapp..

[60] Fedorov A., Beichel R., Kalpathy-Cramer J., Finet J., Fillion-Robin J.C., Pujol S., Bauer C., Jennings D., Fennessy F., Sonka M. (2012). 3D Slicer as an image computing platform for the Quantitative Imaging Network. Magn. Reson. Imaging.

[61] Hanley J.A., McNeil B.J. (1982). The meaning and use of the area under a receiver operating characteristic (ROC) curve. Radiology.

[62] Ohba S., Murayama K., Teranishi T., Kumon M., Nakae S., Yui M., Yamamoto K., Yamada S., Abe M., Hasegawa M. (2023). Three-Dimensional Amide Proton Transfer-Weighted Imaging for Differentiating between Glioblastoma, IDH-Wildtype and Primary Central Nervous System Lymphoma. Cancers.

[63] Mansouri A., Hachem L.D., Mansouri S., Nassiri F., Laperriere N.J., Xia D., Lindeman N.I., Wen P.Y., Chakravarti A., Mehta M.P. (2019). MGMT promoter methylation status testing to guide therapy for glioblastoma: Refining the approach based on emerging evidence and current challenges. Neuro Oncol..

[64] Wang W., Wang M., Jiang H., Wang T., Da R. (2021). BRAFnon-V600E more frequently co-occurs with IDH1/2 mutations in adult patients with gliomas than in patients harboring BRAFV600E but without a survival advantage. BMC Neurol..

[65] Li L., Chen W., Yan Z., Feng J., Hu S., Liu B., Liu X. (2020). Comparative Analysis of Amide Proton Transfer MRI and Diffusion-Weighted Imaging in Assessing p53 and Ki-67 Expression of Rectal Adenocarcinoma. J. Magn. Reson. Imaging.

[66] Jin Y., Xiao W., Song T., Feng G., Dai Z. (2016). Expression and Prognostic Significance of p53 in Glioma Patients: A Meta-analysis. Neurochem. Res..

[67] Sabapathy K. (2015). The Contrived Mutant p53 Oncogene—Beyond Loss of Functions. Front. Oncol..

[68] Sabapathy K., Lane D.P. (2018). Therapeutic targeting of p53: All mutants are equal, but some mutants are more equal than others. Nat. Rev. Clin. Oncol..

[69] Muller P.A., Vousden K.H. (2014). Mutant p53 in cancer: New functions and therapeutic opportunities. Cancer Cell.

[70] Freed-Pastor W.A., Prives C. (2012). Mutant p53: One name, many proteins. Genes. Dev..

[71] Kikuchi S., Nishimura R., Osako T., Okumura Y., Nishiyama Y., Toyozumi Y., Arima N. (2013). Definition of p53 overexpression and its association with the clinicopathological features in luminal/HER2-negative breast cancer. Anticancer Res..

[72] Shifeng T., Yue W., Wen Z., Lihua C., Nan W., Liangjie L., Ailian L. (2023). The value of multimodal functional magnetic resonance imaging in differentiating p53abn from p53wt endometrial carcinoma. Acta Radiol..

[73] Wang Y.Y., Zhang T., Li S.W., Qian T.Y., Fan X., Peng X.X., Ma J., Wang L., Jiang T. (2015). Mapping p53 mutations in low-grade glioma: A voxel-based neuroimaging analysis. AJNR Am. J. Neuroradiol..

[74] Khalid F., Goya-Outi J., Escobar T., Dangouloff-Ros V., Grigis A., Philippe C., Boddaert N., Grill J., Frouin V., Frouin F. (2023). Multimodal MRI radiomic models to predict genomic mutations in diffuse intrinsic pontine glioma with missing imaging modalities. Front. Med..

[75] Li Y., Qian Z., Xu K., Wang K., Fan X., Li S., Jiang T., Liu X., Wang Y. (2018). MRI features predict p53 status in lower-grade gliomas via a machine-learning approach. Neuroimage Clin..

[76] Calabrese E., Rudie J.D., Rauschecker A.M., Villanueva-Meyer J.E., Clarke J.L., Solomon D.A., Cha S. (2022). Combining radiomics and deep convolutional neural network features from preoperative MRI for predicting clinically relevant genetic biomarkers in glioblastoma. Neurooncol Adv..

[77] Durmo F., Rydhog A., Testud F., Latt J., Schmitt B., Rydelius A., Englund E., Bengzon J., van Zijl P., Knutsson L. (2020). Assessment of Amide proton transfer weighted (APTw) MRI for pre-surgical prediction of final diagnosis in gliomas. PLoS ONE.

[78] Zhou J., Zaiss M., Knutsson L., Sun P.Z., Ahn S.S., Aime S., Bachert P., Blakeley J.O., Cai K., Chappell M.A. (2022). Review and consensus recommendations on clinical APT-weighted imaging approaches at 3T: Application to brain tumors. Magn. Reson. Med..

[79] Herz K., Mueller S., Perlman O., Zaitsev M., Knutsson L., Sun P.Z., Zhou J., van Zijl P., Heinecke K., Schuenke P. (2021). Pulseq-CEST: Towards multi-site multi-vendor compatibility and reproducibility of CEST experiments using an open-source sequence standard. Magn. Reson. Med..

