# Supplementary material for: Role of Amide Proton Transfer Weighted MRI in Predicting MGMTp Methylation Status, p53-Status, Ki-67 Index, IDH-Status, and ATRX Expression in WHO Grade 4 High Grade Glioma
Source: Tomography. 2025 May 31;11(6):64. doi: 10.3390/tomography11060064 (PMC12196788; doi:10.3390/tomography11060064)
Supplement: Supplementary file 1 [file tomography-11-00064-s001.zip › tomography-3546020-supplementary.pdf]

tomography

MDPI

Supplementary Material

Table S1. Demographics, mutation status, for a total of 36 Glioblastoma patients after re-classification according to WHO CNS5 /2021 classification.

Histological or demographic features		Count
Biological Sex	Female	10
	Male	26
ATXR-Wildtype	No	3
	Yes	33
IDH-Wildtype	No	0
	Yes	36
Ki-67 index > 10 % (%)	No	9
	Yes	26
MGMT Promoter Methylation**	No	17
	Yes	16
p53-normal	No	12
	Yes	24
Age at APTw MRI exam (years)	Overall (y)	59
	Male vs. Female (y)	58 vs 62
	MGMTp meth vs. nonmeth (y)	63 vs 56
	ATRX-wt vs. ATRX-mut (y)	61 vs 43
	IDH-wt vs. IDH-mut (y)	59 vs 38*
	Ki-67-low vs. Ki-67-high (y)	59 vs 59
	P53-wt vs p53-overexpressed	59 vs 59

*P-value = 0.001 and denotes a statistically significant difference.
**Missing cases in total: MGMT promoter methylation (n = 3), Ki-67 index (n = 1).

Table S2. APTw% signal (group average) across ATRX-, MGMTp methylation-, IDH- and Ki-67-status, p53 status (after re-classification according to the WHO CNS 5/2021 classification for Glioblastoma (n = 36 IDH-wt)).

	mean-APTw %[SD]	median-APTw % [SD]	max-APTw % [SD]
ATRX -mut (n = 3)	1.35 [0.17]	1.35 [0.21]	5.15 [0.90]
ATRX-wt (n = 33)	1.64 [0.80]	1.66 [0.80]	4.97 [2.42]
IDH-wt (n = 36)	1.61 [0.77]	1.64 [0.77]	4.99 [2.33]
MGMTp methylated (n = 16)**	1.50 [0.58]	1.59 [0.62]	4.02 [1.64]
MGMTp non-methylated. (n = 17)**	1.71 [0.93]	1.70 [0.91]	5.78 [2.72]
Ki-67 low (n = 9)***	1.78 [0.57]	1.68 [0.47]	5.61 [2.80]
Ki-67 high (n = 26)***	1.55 [0.85]	1.58 [0.84]	4.78 [2.20]
p53-normal (n = 24)	1.86* [0.80]	1.87* [0.80]	5.35 [2.37]
p53-overexpressed (n = 12)	1.11* [0.39]	1.16* [0.39]	4.27 [2.16]

*p-value < 0.05 denoting statistically significant difference.

Missing cases MGMTpmeth status (n = 3), * Ki-67 index status missing cases (n = 1)

Table S3. Spatial distribution# of overexpressed p53, normal p53, non-methylated MGMTp, and methylated MGMTp in different brain regions in 36 Glioblastoma patients after re-classification according to WHO CNS /2021 classification.

Anatomical Lesion Location	Frontal		Temporal		Parietal	
Glioblastoma and molecular characteristic	Sin	Dx	Sin	Dx	Sin	Dx
Overexpressed p53	3	1	3	1	3	2
Normal p53	4	4	4	4	3	5
Non-methylated MGMTp	4	2	2	3	6	2
Methylated MGMTp	2	3	4	2		3

The frequency is presented for each type in frontal, temporal, and parietal regions, further categorized by hemisphere with 'sin' representing the left brain hemisphere and 'dx' representing the right brain hemisphere. 3 patients crossed the midline and are not accorded and classified for in the table.

Table S4. ROC analysis of mean- and median- APTw% signal and their combination through logistic regression for distinguishing p53 normal expressed Glioblastoma vs p53 overexpressed in 36 Glioblastoma patients after re-classification according to WHO CNS /2021 classification

	Area under the curve (AUC)	p-value	95% Confidence Interval		Cutoff/ Sensitivity/ Specificity
			Lower Bound	Upper Bound	
Mean-APTw	0.823	0.002	0.69	0.96	1.43% / 75% / 83.3.0%
Median-APTw	0.769	0.009	0.62	0.92	1.42% / 70.8% / 66.7%
Mean+median-APTw	0.819	0.002	0.68	0.96	0.66* / 75% / 75%

*Probabilistic value provided by the logistic regression model and not APTw% signal value. GBMs with p53 normal n = 24, GBMs with p53 overexpressed n= 12

Table S5. APTw% signal (group average) across ATRX-, MGMTp methylation-, IDH- and Ki-67-status, p53 status for high grade glioma.

	mean-APTw % [SD]	Median-APTw %[SD]	max-APTw % [SD]
ATRX -mut (n = 6)	1.30 [0.18]	1.28 [0.21]	5.22 [1.24]
ATRX-wt (n = 36)	1.61 [0.78]	1.63 [0.77]	4.76 [2.43]
IDH-mut (n = 6)	1.26 [0.15]	1.24 [0.17]	3.84 [1.94]
IDH-wt (n = 36)	1.61 [0.77]	1.64 [0.77]	4.99 [2.33]
MGMTp methylated (n = 21)	1.46 [0.51]	1.51 [0.56]	3.98* [1.72]
MGMTp non-methylated (n =17)	1.71 [0.93]	1.70 [0.91]	5.78* [2.72]
Ki-67 low (n = 11)	1.72 [0.54]	1.63 [0.44]	5.21 [2.74]
Ki-67 high (n = 30)	1.51 [0.80]	1.52 [0.80]	4.69 [2.19]
p53-normal (n = 26)	1.81* [0.79]	1.83* [0.79]	5.12 [2.42]
p53-overexpressed (n = 16)	1.15* [0.35]	1.18* [0.35]	4.35 [2.10]

*p-value < 0.05 denoting statistically significant difference.

Table S6. The molecular and genetic profiles for each patient along with type of surgery for obtaining tissue that was sent for histopathological analysis and histological diagnosis according to both the WHO 2016 CNS tumor classification and the most recent one from 2021.

Patient	MGMTp methylation	IDH wildtype	P53 preserved	ATRX mutation	Ki-67 index above 10%	Histology WHO 2016	Histology WHO 2021	Type of surgery for histology
1	Yes	No	No	Yes	No	HGG grade 4	Astrocytoma grade 4	Resection
2	X	Yes	No	No	Yes	HGG grade 4	Glioblastoma IDHwt grade 4	Resection
3	X	No	No	Yes	Yes	HGG grade 4	Astrocytoma grade 4	Resection
4	No	Yes	No	Yes	Yes	HGG grade 4	Glioblastoma IDHwt grade 4	Resection
5	Yes	Yes	No	No	Yes	HGG grade 4	Glioblastoma IDHwt grade 4	Resection
6	No	Yes	No	No	No	HGG grade 4	Glioblastoma IDHwt grade 4	Resection
7	Yes	Yes	No	No	Yes	HGG grade 4	Glioblastoma IDHwt grade 4	Resection
8	No	Yes	No	No	Yes	HGG grade 4	Glioblastoma IDHwt grade 4	Resection
9	Yes	No	No	Yes	Yes	HGG grade 4	Astrocytoma grade 4	Resection
10	Yes	Yes	No	No	Yes	HGG grade 4	Glioblastoma IDHwt grade 4	Resection
11	Yes	Yes	No	No	Yes	HGG grade 4	Glioblastoma IDHwt grade 4	Biopsy
12	Yes	Yes	No	No	Yes	HGG grade 4	Glioblastoma IDHwt grade 4	Biopsy
13	Yes	No	No	No	Yes	HGG grade 4	Astrocytoma grade 4	Resection
14	Yes	Yes	No	No	Yes	HGG grade 4	Glioblastoma IDHwt grade 4	Resection

15	Yes	Yes	No	No	Yes	HGG grade 4	Glioblastoma IDHwt grade 4	Biopsy
16	No	Yes	No	No	Yes	HGG grade 4	Glioblastoma IDHwt grade 4	Resection
17	Yes	Yes	Yes	No	Yes	HGG grade 4	Glioblastoma IDHwt grade 4	Biopsy
18	X	Yes	Yes	No	Yes	HGG grade 4	Glioblastoma IDHwt grade 4	Biopsy
19	X	Yes	Yes	No	No	HGG grade 4	Glioblastoma IDHwt grade 4	Biopsy
20	Yes	Yes	Yes	No	Yes	HGG grade 4	Glioblastoma IDHwt grade 4	Resection
21	Yes	Yes	Yes	No	Yes	HGG grade 4	Glioblastoma IDHwt grade 4	Resection
22	No	Yes	Yes	Yes	Yes	HGG grade 4	Glioblastoma IDHwt grade 4	Resection
23	No	Yes	Yes	No	Yes	HGG grade 4	Glioblastoma IDHwt grade 4	Resection
24	No	Yes	Yes	No	Yes	HGG grade 4	Glioblastoma IDHwt grade 4	Resection
25	No	Yes	Yes	No	Yes	HGG grade 4	Glioblastoma IDHwt grade 4	Resection
26	No	Yes	Yes	No	No	HGG grade 4	Glioblastoma IDHwt grade 4	Biopsy
27	Yes	Yes	Yes	No	No	HGG grade 4	Glioblastoma IDHwt grade 4	Biopsy
28	Yes	No	Yes	No	Yes	HGG grade 4	Astrocytoma grade 4	Resection
29	No	Yes	Yes	No	Yes	HGG grade 4	Glioblastoma IDHwt grade 4	Resection
30	No	Yes	Yes	Yes	Yes	HGG grade 4	Glioblastoma IDHwt grade 4	Resection
31	Yes	Yes	Yes	No	Yes	HGG grade 4	Glioblastoma IDHwt grade 4	Resection

32	Yes	Yes	Yes	No	No	HGG grade 4	Glioblastoma IDHwt grade 4	Resection
33	No	Yes	Yes	No	No	HGG grade 4	Glioblastoma IDHwt grade 4	Resection
34	Yes	No	Yes	No	No	HGG grade 4	Astrocytoma grade 4	Resection
35	Yes	Yes	Yes	No	X	HGG grade 4	Glioblastoma IDHwt grade 4	Resection
36	Yes	Yes	Yes	No	No	HGG grade 4	Glioblastoma IDHwt grade 4	Resection
37	No	Yes	Yes	No	Yes	HGG grade 4	Glioblastoma IDHwt grade 4	Biopsy
38	Yes	Yes	Yes	No	Yes	HGG grade 4	Glioblastoma IDHwt grade 4	Resection
39	No	Yes	Yes	No	No	HGG grade 4	Glioblastoma IDHwt grade 4	Resection
40	No	Yes	Yes	No	Yes	HGG grade 4	Glioblastoma IDHwt grade 4	Biopsy
41	No	Yes	Yes	No	No	HGG grade 4	Glioblastoma IDHwt grade 4	Resection
42	No	Yes	Yes	No	Yes	HGG grade 4	Glioblastoma IDHwt grade 4	Resection

Note, all IDH mutations were solely IDH1mutations.
